# Health care utilization among patients with oesophageal and gastric cancer: the impact of initial treatment strategy and assignment of a contact nurse

**DOI:** 10.1186/s12913-021-07042-7

**Published:** 2021-09-27

**Authors:** Karin Dalhammar, Marlene Malmström, Magnus Sandberg, Dan Falkenback, Jimmie Kristensson

**Affiliations:** 1 Institute for Palliative Care, Lund University and Region Skåne, Lund, Sweden; 2 Department of Health Sciences, Faculty of Medicine, Lund University, Lund, Sweden; 3 Department of Surgery, Skåne University Hospital, Lund, Sweden; 4 Department of Clinical Sciences Lund, Faculty of Medicine, Lund University, Lund, Sweden

**Keywords:** Health care utilization, Treatment strategy, Oesophageal cancer, Gastric cancer, Palliative care, Contact nurse

## Abstract

**Background:**

Patients diagnosed with oesophageal and gastric cancer face a poor prognosis and numerous challenges of symptom management, lifestyle adjustments and complex treatment regimens. The multifaceted care needs and rapid disease progression reinforce the need for proactive and coherent health care. According to the national cancer strategy, providing coherent health care and palliative support is an area of priority. More knowledge is needed about health care utilization and the characteristics of the health care service in order to understand the readiness, accessibility and quality of current health care. The aim of this study was to describe individuals’ health care use from the time of treatment decision until death, and investigate the impact of the initial treatment strategy and assignment of a contact nurse (CN) on health care use among patients with oesophageal and gastric cancer.

**Methods:**

This population-based cohort study included patients who died from oesophageal and gastric cancer in Sweden during 2014–2016. Through linking data from the National Register for Oesophageal and Gastric Cancer, the National Cause of Death Register, and the National Patient Register, 2614 individuals were identified. Associations between the initial treatment strategy and CN assignment, and health care use were investigated. Adjusted incidence rate ratios (IRRs) with 95% confidence intervals (CIs) were calculated using Poisson regression.

**Results:**

Patients receiving palliative treatment and those receiving no tumour-directed treatment had a higher IRR for unplanned hospital stays and unplanned outpatient care visits compared with patients who received curative treatment. Patients receiving no tumour-directed treatment also had a lower IRR for planned hospital stays and planned outpatient care visits compared with patients given curative treatment. Compared with this latter group, patients with palliative treatment had a higher IRR for planned outpatient care visits. Patients assigned a CN had a higher IRR for unplanned hospital stays, unplanned outpatient care visits and planned outpatient care visits, compared with patients not assigned a CN.

**Conclusions:**

A palliative treatment strategy and no tumour-directed treatment were associated with higher rates of unplanned health care compared with a curative treatment strategy, suggesting that a proactive approach is imperative to ensure quality palliative care.

## Introduction

Despite ongoing medical care and complex treatment regimens, patients diagnosed with oesophageal and gastric cancer face a poor prognosis and numerous challenges related to symptom management, emotional distress and lifestyle adjustments [1–3]. The multifaceted care needs and the rapid disease progression necessitate involvement of multiple health care providers with a high degree of proactivity, continuity and integration to achieve high-quality care. A palliative care approach focusing on individualized care, interprofessional care coordination and anticipatory symptom management is therefore of utmost importance. However, health care utilization among patients with oesophageal and gastric cancer is sparsely investigated from a broader timeline perspective, especially with regard to the initial treatment regimen. Such knowledge is essential to gain a deeper understanding of the readiness, accessibility and quality of current health care service.

Oesophageal and gastric cancer are the sixth and third leading causes of cancer death worldwide [4]. The overall prognosis is poor, with a 5-year survival estimate of 20–30% [5]. In Sweden, about 1300 people are diagnosed annually and the diseases cause about 1000 deaths each year [6]. The patients’ medical treatment and care contacts may be more or less extensive depending on whether the treatment aim is curative or palliative. About 40% of newly diagnosed patients can be offered treatment with curative intent, usually surgery alone or in combination with neoadjuvant therapy [7, 8]. However, the recurrence rate is high (30–67%) within the first postoperative year and the procedure is associated with extensive care needs due to postoperative complications or treatment-related side effects [9, 10]. Individuals with severe comorbidity and locally advanced or metastatic cancer represent approximately 60% of patients and are usually offered palliative chemotherapy and/or palliative surgery to extend life, relieve symptoms and maintain quality of life [7, 8]. Patients with any type of tumour directed therapy (curative and palliative) are followed up in specialized outpatient clinics, according to national guidelines, to support the recovery process and reduce the risk of complications during and after treatment, while patients with no tumour directed treatment are referred to primary care.

Regardless of whether the initial treatment strategy is curative or palliative, patients are likely to suffer from a significant long-term decrease in quality of life and multiple symptoms related to treatment and disease progression, such as weight loss, nausea and pain [2, 3]. Psychosocial distress including anxiety, depression and fatigue has also been reported [1].

Patients with oesophageal and gastric cancer are therefore in need of social and medical support from a wide variety of health care providers throughout their illness trajectory. However, having multiple health care providers can pose a risk of fragmented care, with consequences such as worse quality of care and increased acute care use [11]. Highly coordinated health care is therefore essential from both an organizational and a patient perspective. The characteristics of the health care provided for these patients are not well investigated, especially with regard to the potential impact of the initial treatment strategy. The characteristics of the health care use, following a diagnosis of oesophageal and gastric cancer, may differ according to the patients’ initial treatment strategy. Patients with any type of tumour-directed treatment (palliative and curative) have a defined chain of care that includes treatment and follow up within specialized care, while patients who receive no tumour directed therapy lack thereof. In this sense, patients with no tumour directed therapy have no clear link to the specialized health care system, which might have implications for the characteristics of their health care use.

To ensure continuity of care and proactive support for patients with life-limiting illness and complex care needs, the American Society of Clinical Oncology (ASCO) recommends integration of a palliative approach within 8 weeks of diagnosis [12]. The aim of a palliative approach is to optimize the quality of life for patients and their families.

A palliative care approach has gained increased attention across multiple disciplines such as oncology and surgery [13, 14]. In 2009, the Swedish government adopted a national cancer strategy to improve cancer care, which highlights palliative care as a focus area. The strategy further emphasizes continuity and easy access as key quality factors in the cancer care process. To ensure coherent health care, it specifically suggests that all patients should be offered the services of a contact nurse (CN), at the time of diagnosis, who has the overall responsibility to coordinate and maintain person-centred care while collaborating with other health care professionals such as e.g. palliative care specialists throughout the cancer trajectory [15]. However, there is a paucity of studies investigating the potential impact of CN support on patients’ health care utilization. Such knowledge could provide valuable insight into the CN’s potential to facilitate coherent and accessible health care.

Health care utilization is widely used as an indicator for quality of palliative care. According to the ASCO, high care quality is characterized by low frequency of ED visits and hospitalizations and high rate of hospice enrolment [16]. Despite the recognized need for proactivity, studies indicate that acute health care is common among patients with advanced cancer [17, 18]. A cross-national study on patients dying from cancer in seven developed countries reports that 44–64% were admitted to acute care hospitals and 28–58% visited the ED in the final month of life [19]. In one study focusing on oesophageal and gastric cancer, nearly 50% of patients were admitted to acute hospital care in the last month of life [20] while a Korean study reports that 39% of patients with gastric cancer visited the ED more than once during the final month of life [21]. Although the care needs may be more extensive in the later stage of disease, this is still incongruent with a palliative care approach focusing on proactive support. There is a great need to identify factors associated with acute health care use and to address this incongruence.

Previous research indicates that tumour-directed treatment in the final months of life is associated with increased ED use [22]. Studies also indicate that health care use, during the final month of life, differs by initial treatment strategy [23, 24]. Van den Block et al. demonstrated that a curative treatment strategy increased the odds of hospitalization during the last 3 months of life fivefold compared with a palliative strategy [24]. However, little is known about the impact of the initial treatment strategy on health care use among patients with oesophageal and gastric cancer and there is a paucity of studies that investigate health care use from a broader timeline perspective. Given the low likelihood of cure among patients with oesophageal and gastric cancer and the increasing policy attention to early integrated palliative care, there is a great need to examine the association between the initial treatment strategy and the pattern of health care use from a broader timeline perspective. Such knowledge could provide valuable insight into the ability of health care to deliver quality palliative care, and could also inform future interventions and health resource allocations to those who may benefit from them most.

The aim of this study was therefore to describe health care use from the time of treatment decision until death and investigate the impact of the initial treatment strategy and assignment of a contact nurse on health care use among patients with oesophageal and gastric cancer.

## Methods

### Design

This study was a population-based cohort study.

### Study population

The sample comprised 2636 individuals who died between 1 January 2014 and 31 December 2016 in Sweden, with oesophageal and gastric cancer as the underlying cause of death. They were identified by means of two registers: the National Register for Oesophageal and Gastric Cancer (NREV) and the National Cause of Death Register.

### Data collection

Data were collected from the three registers. The NREV is a national quality register comprising information about diagnostics, clinical manifestations, outcome of surgical treatment and follow-up of oesophageal and gastric cancer. The completeness of the registration data at a national level is > 95% [25]. From the NREV, all patients were identified and data about date of diagnosis, tumour site, histology, performance status according to the scale of the Eastern Cooperative Oncology Group (0–5, with a lower value representing better function) [26], clinical M stage (which refers to whether the cancer has metastasized [M1] or not [M0]), CN assignment (which refers to whether the patient, at the time of diagnosis, was assigned a CN or not) and initial medical treatment strategy, curative (tumour-directed treatment such as surgery/chemotherapy/ radiotherapy with a curative intent) or palliative (tumour-directed treatment such as surgery/chemotherapy/radiotherapy with a palliative intent) or no tumour-directed therapy (palliative without treatment), were extracted.

The National Cause of Death Register is held by the National Board of Health and Welfare and covers 99% of all deaths in Sweden [27]. From the National Cause of Death Register, the date of death and underlying cause of death, according to the International Statistical Classification of Diseases and Related-Health Problems, Tenth Revision (ICD-10) [28], were extracted and linked to the NREV; only data on individuals with oesophageal and gastric cancer as the underlying cause of death were kept.

The National Patient Register (NPR) is also held by the National Board of Health and Welfare and comprises information about public and private, psychiatric and somatic inpatient and specialist outpatient health care. The register does not contain information about primary health care. Inpatient and outpatient coverage is approximately 99 and 87%, respectively [29]. The cause to care is registered as one main diagnosis and up to 20 secondary diagnoses, according to the ICD-10. From the NPR, data about the date of somatic planned (pre-booked)/unplanned (unanticipated, unscheduled) inpatient and specialist outpatient visits, bed days and the main cause for seeking care were extracted and linked to the corresponding patient.

**Fig. 1:**
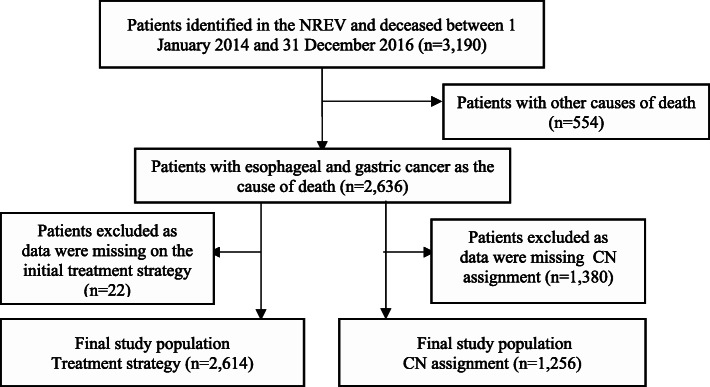
Flow chart of inclusion in analysis of initial treatment strategy and contact nurse (CN) assignment

In the linked dataset, NREV data about the initial treatment strategy were missing for 22 patients and about CN assignment for 1380 patients. These exclusions left a total of 2614 persons who were included in the analysis of the initial treatment strategy and 1256 persons who were included in the analysis of CN assignment (Fig. 1).

### Data analysis

The sample (*n* = 2614) were categorized into three pre-defined categories according to the initial treatment strategy: curative, palliative, and no tumour-directed treatment.

Baseline data on demographic and clinical characteristics were analysed with descriptive and analytical statistics. Differences were calculated using analysis of variance (ANOVA) for continuous variables, chi-square test or Fisher’s exact test for categorical variables and Kruskal-Wallis test for skewed continuous data.

In the total number of hospital stays, the three most common ICD chapters and categories were determined and the median number of specialist outpatient care visits, hospital stays (planned, unplanned, total) and bed days were calculated for each treatment strategy group. Differences were analysed using the Kruskal-Wallis test, as the data were skewed. The Dunn-Bonferroni test was used for pairwise multiple comparisons.

The outcome variables were created by aggregating the total number of planned/unplanned inpatient and specialist outpatient somatic care visits for each individual.

Poison regressions, providing incidence rate ratios (IRRs) with 95% confidence intervals (CIs), were performed to assess the impact of the initial treatment strategy and assignment of a CN on each health care utilization outcome. For the analysis of initial treatment strategy, the category “curative” was used as a reference category; for the analysis of CN assignment, the category “No CN assignment” was used as reference category. An offset variable, log(number of days from treatment decision to death), was used in the models to account for variation in exposure time. Possible confounders that were taken into account in the statistical models were: sex, age (categorized into quartiles: < 65, 66–72, 73–79, ≥80 years old), M stage, and performance status. The statistical analysis of CN assignment was also adjusted for initial treatment strategy.

All statistical analyses were performed using IBM SPSS statistics version 25 (IBM Corp., Armonk, N.Y., USA). A *p*-value of < 0.05 was used to define statistical significance.

### Ethical approval

The study was conducted in accordance with the act for ethical review of research involving humans, SFS 2003:460, and ethical approval was obtained from the Regional Ethics Review Board in Lund (REC number: 2018/03, 2018/270, 2020/03596). A waiver for informed consent was obtained by the Regional Ethics Review Board.

## Results

Of the 2614 individuals included, 1773 (67.8%) were men. The mean age at diagnosis was 71.4 years (standard deviation (SD) ±11.5) (Table 1). Adenocarcinoma and squamous cell carcinoma accounted for 76.8 and 16.6%, respectively, of all cancers. Among all patients, 1505 (57.6%) had a tumour originating in the oesophagus, 1092 (42.2%) had distant metastases (M1) at the time of diagnosis, and 907 (36.3%) had a performance status score of 1. The median survival time was 7.0 (range 2.0–16.0) months. In total, 1278 (48.9%) received a palliative treatment strategy, 877 (33.6%) curative treatment, and 459 (17.6%) no tumour-directed treatment*.*

**Table 1:** Baseline demographic and clinical characteristics, by initial treatment strategy and contact nurse (CN) assignment

	Initial treatment strategy					CN assignment			
	Total n = 2614	Curative *n* = 877 (33.6%)	Palliative *n* = 1278 (48.9%)	No tumour-directed treatment *n* = 459 (17.6%)	p-value	Total *n* = 1256	Yes *n* = 955 (76.0%)	No *n* = 301 (24.0%)	p-value
Age, yrs. (mean ± SD)	71.4 ± 11.5	67.8 ± 10.4	71.3 ± 11.5	78.7 ± 10.2	< 0.001	72.6 ± 11.29	72.2 ± 11.0	73.9 ± 12.0	0.023
Men, n (%)	1773 (67.8)	614 (70.0)	887 (69.4)	272 (59.3)	< 0.001	835 (66.5)	641 (67.1)	194 (64.5)	0.216
Women, n (%)	841 (32.2)	263 (30.0)	391 (30.6)	187 (40.7)		421 (33.5)	314 (32.9)	107 (35.5)	
**Survival time from diagnosis**	*n* = 2605^1^					*n* = 1251^2^			
Months (median, Q1–Q3)	7.0 (2.0–16.0)	15.0 (8.0–27.0)	5.0 (2.0–11.0)	1.5 (0.0–5.0)	< 0.001	4.0 (1.0–9.0)	4.0 (2.0–9.0)	3.0 (1.0–10.0)	0.011
**Initial treatment strategy**						*n* = 1253^3^			< 0.001
Curative	–	–	–	–		277 (22.1)	232 (24.3)	45 (15.0)	
Palliative	–	–	–	–		708 (56.5)	566 (59.4)	142 (47.3)	
No tumour-directed treatment	–	–	–	–		268 (21.4)	155 (16.3)	113 (37.7)	
**Histological type, n (%)**	*n* = 2595^4^				< 0.001	*n* = 1251^5^			0.268
Adenocarcinoma	1993 (76.8)	663 (75.9)	967 (76.1)	363 (80.3)		946 (75.6)	709 (74.6)	237 (78.7)	
Non-differentiated	42 (1.6)	10 (1.1)	25 (2.0)	7 (1.5)		25 (2.0)	21 (2.2)	4 (1.3)	
Squamous cell carcinoma	430 (16.6)	163 (18.7)	224 (17.6)	43 (9.5)		214 (17.1)	172 (18.1)	42 (14.0)	
Other	130 (5.0)	37 (4.2)	54 (4.3)	39 (8.6)		66 (5.3)	48 (5.1)	18 (6.0)	
**M stage at diagnosis, n (%)**	*n* = 2586^6^				< 0.001	*n* = 1241^7^			0.320
M0	1494 (57.8)	835 (96.2)	459 (36.1)	200 (44.6)		610 (49.2)	469 (49.6)	141 (47.8)	
M1	1092 (42.2)	33 (3.8)	811 (63.9)	248 (55.4)		631 (50.8)	477 (50.4)	154 (52.2)	
**Performance status, n (%)**	*n* = 2501^8^				< 0.001	*n* = 1247^9^			0.090
0	671 (26.8)	410 (48.7)	241 (19.7)	20 (4.6)		268 (21.5)	209 (22.0)	59 (19.8)	
1	907 (36.3)	340 (40.4)	478 (39.0)	89 (20.5)		348 (27.9)	264 (27.8)	84 (28.2)	
2	617 (24.7)	81 (9.6)	368 (30.0)	168 (38.7)		384 (30.8)	303 (31.9)	81 (27.2)	
3	261 (10.4)	11 (1.3)	128 (10.4)	122 (28.1)		214 (17.2)	152 (16.0)	62 (20.8)	
4	45 (1.8)	0 (0.0)	10 (0.8)	35 (8.1)		33 (2.6)	21 (2.2)	12 (4.0)	
**Site of primary tumour**	*n* = 2612^10^				< 0.001	*n* = 1255^11^			0.005
Oesophageal cancer	1505 (57.6)	516 (58.8)	814 (63.7)	175 (38.2)		738 (58.8)	581 (60.9)	157 (52.2)	
Gastric cancer	1107 (42.4)	361 (41.2)	463 (36.3)	283 (61.8)		517 (41.2)	373 (39.1)	144 (47.8)	

^1^9 missing; ^2^5 missing; ^3^3 missing; ^4^19 missing; ^5^5 missing; ^6^28 missing; ^7^15 missing; ^8^113 missing; ^9^9 missing; ^10^2 missing; ^11^1 missing. M0 = the cancer has not metastasized; M1 = the cancer has metastasized; Q1 = first quartile; Q3 = third quartile; SD = standard deviation

### Health care utilization, by initial treatment strategy and contact nurse assignment

The median number of total hospital stays was higher among patients with a curative treatment strategy compared with patients with a palliative treatment strategy and patients with no tumour-directed treatment (5.0 [3.0–8.0] v 3.0 [1.0–4.0] and 1.0 [1.0–2.0]). Patients with a curative treatment strategy also had a higher median number of bed days, in comparison with patients with a palliative treatment strategy and patients with no tumour-directed treatment (46.0 [28.0–70.0] v 20.0 [8.0–36.0] and 8.0 [2.0–19.0]), and a higher median number of specialized outpatient care visits (16.0 [9.0–28.0] v 7.0 [3.0–14.0] and 1.0 [0.0–3.0]) in comparison with patients with a palliative treatment strategy and patients with no tumour-directed treatment (Table 2).

**Table 2:** Health care use, by initial treatment strategy and contact nurse (CN) assignment

	Initial treatment strategy					CN assignment			
	Total n = 2614	Curative n = 877	Palliative n = 1278	No tumour-directed treatment n = 459	p-value^1^	Total n = 1256	Yes n = 955 (76.0%)	No n = 301 (24.0%)	p-value^2^
**Hospital stays, median (Q1–Q3)**									
Unplanned	2.0 (1.0–4.0)	3.0 (2.0–5.0)	2.0 (1.0–3.0)	1.0 (1.0–2.0)	**< 0.001**^1^	2.0 (1.0–4.0)	2.0 (1.0–3.0)	1.0 (1.0–2.0)	**0.007**
Planned	1.0 (0.0–2.0)	2.0 (1.0–3.0)	1.0 (0.0–1)	0.0 (0.0–1.0)	**< 0.001**^1^	1.0 (0.0–2.0)	1.0 (0.0–2.0)	0.0 (0.0–1.0)	**0.024**
Total (unplanned and planned)	3.0 (2.0–6.0)	5.0 (3.0–8.0)	3.0 (1.0–4.0)	1.0 (1.0–2.0)	**< 0.001**^1^	4.0 (2.0–7.0)	3.0 (1.0–4.0)	2.0 (1.0–4.0)	**< 0.001**
Bed days	24.0 (10.0–47.0)	46.0 (28.0–70.0)	20.0 (8.0–36.0)	8.0 (2.0–19.0)	**< 0.001**^1^	32.0 (14.0–56.0)	19.0 (8.0–37.0)	14.0 (4.0–29.0)	**< 0.001**
**Visits, specialist outpatient care, median (Q1–Q3)**									
Unplanned	1.0 (0.0–3.0)	2.0 (1.0–4.0)	1.0 (0.0–2.0)	0.0 (0.0–1.0)	**< 0.001**^1^	1.0 (0.0–2.0)	1.0 (0.0–2.0)	0.0 (0.0–2.0)	**< 0.001**
Planned	6.0 (2.0–14.0	13.0 (7.0–23.5)	5.0 (2.0–12.0)	1.0 (0–2.0)	**< 0.001**^1^	4.0 (1.0–10.0)	4.0 (1.0–10.0)	2.0 (0.0–10.0)	**< 0.001**
Total (unplanned and planned)	8.0 (3.0–17.0)	16.0 (9.0–28.0)	7.0 (3.0–14.0)	1 (0.0–3.0)	**< 0.001**^1^	5.0 (2.0–13.0)	6.0 (2.0–13.0)	3.0 (0.0–12.0)	**< 0.001**

^1^Post-hoc analysis showed significant differences between all groups; ^2^Mann-Whitney U-test. Q1 = first quartile; Q3 = third quartile

The three most common primary diagnosis groups among the 4845 (51.0%) hospital stays of patients with a curative treatment strategy were “neoplasms”, *n* = 2869 (59.2%), “factors influencing health status and contact with health services”, *n* = 675 (13.9%), and “symptoms, signs and abnormal clinical and laboratory findings not elsewhere classified”, *n* = 416 (8.6%). The most common primary diagnosis groups among the 3816 (40.2%) hospital stays among patients with a palliative treatment strategy were “neoplasms”, *n* = 2615 (68.5%), followed by “symptoms, signs and abnormal clinical and laboratory findings not elsewhere classified”, *n* = 347 (9.1%), and “factors influencing health status and contact with health services”, *n* = 282 (7.4%). For patients with no tumour-directed therapy, *n* = 841 (8.9%), the most common primary diagnosis groups were “neoplasms”, *n* = 662 (78.7%), followed by “diseases of the respiratory system”, *n* = 45 (5.4%), and “symptoms, signs and abnormal clinical and laboratory findings not elsewhere classified”, *n* = 41 (4.9%) (Table 3).

**Table 3:** Primary diagnosis according to ICD-10 by initial treatment strategy and total number of hospital stays

Total, ***n*** = 9502^**1**^ Chapter, n (%)^2^ Categories, n (%)^3^	Curative, n (%) 4845 (51.0) Chapter, n (%)^2^ Categories, n (%)^3^	Palliative, n (%) 3816 (40.2) Chapter, n (%)^2^ Categories, n (%)^3^	No tumour-directed treatment, n (%) 841 (8.9) Chapter, n (%)^2^ Categories, n (%)^3^
**Neoplasms (C/D), 6146 (64.7)**	**Neoplasms (C/D), 2869 (59.2)**	**Neoplasms (C/D), 2615 (68.5)**	**Neoplasms (C/D), 662 (78.7)**
1. Malignant neoplasm of stomach, (C16) 2800 (45.6)	1. Malignant neoplasm of stomach (C16), 1251 (43.6)	1. Malignant neoplasm of oesophagus (C15), 1248 (47.7)	1. Malignant neoplasm of stomach (C16), 416 (62.8)
2. Malignant neoplasm of oesophagus (C15), 2585 (42.1)	2. Malignant neoplasm of oesophagus (C15), 1149 (40.0)	2. Malignant neoplasm of stomach (C16), 1133 (43.3)	2. Malignant neoplasm of oesophagus (C15), 188 (28.4)
3. Malignant neoplasm of other and ill-defined sites (C78), 314 (5.1)	3. Malignant neoplasm of other and ill-defined sites (C78), 205 (7.1)	3. Malignant neoplasm of other and ill-defined sites (C78), 101 (3.9)	3. Neoplasm of uncertain or unknown behaviour of oral cavity and digestive organs (D37), 20 (3.0)
**Factors influencing health status and contact with health services (Z), 982 (10.3)**	**Factors influencing health status and contact with health services (Z), 675 (13.9)**	**Symptoms, signs and abnormal clinical and laboratory findings not elsewhere classified (R), 347 (9.1)**	**Diseases of the respiratory system (J), 45 (5.4)**
1. Other medical care (Z51), 754 (76.8)	1. Other medical care (Z51), 505 (74.8)	1. Dysphagia (R13), 59 (17.0)	1. Bacterial pneumonia not elsewhere classified (J15), 11 (24.4)
2. Other surgical follow-up care (Z48), 100 (10.2)	2. Other surgical follow-up care (Z48), 88 (13.0)	2. Ascites (R18), 51 (14.7)	2. Other chronic obstructive pulmonary disease (J44), 11 (24.4)
3. Personal history of malignant neoplasm (Z85), 49 (5.0)	3. Personal history of malignant neoplasm (Z85), 43 (6.4)	3. Nausea and vomiting (R11), 44 (12.7)	3. Pneumonia, organism unspecified (J18), 9 (20)
**3. Symptoms, signs and abnormal clinical and laboratory findings not elsewhere classified (R), 804 (8.5)**	**3. Symptoms, signs and abnormal clinical and laboratory findings not elsewhere classified (R), 416 (8.6)**	**3. Factors influencing health status and contact with health services (Z), 282 (7.4)**	**3. Symptoms, signs and abnormal clinical and laboratory findings not elsewhere classified (R), 41 (4.9)**
1. Nausea and vomiting (R11), 116 (14.4)	1. Nausea and vomiting (R11), 63 (15.1)	1. Other medical care (Z51), 241 (85.5)	1. Nausea and vomiting (R11), 9 (22.0)
2. Fever of other and unknown origin (R50), 108 (13.4)	2. Abdominal and pelvic pain (R10), 62 (14.9)	2. Other surgical follow-up care (Z48), 10 (3.5)	2. Abnormalities of breathing (R06), 6 (14.6)
3. Dysphagia (R13), 105 (13.1)	3. Fever of other and unknown origin (R50), 62 (10.1)	3. Adjustment and management of implanted device (Z45), 8 (2.8)	3. Abdominal and pelvic pain (R10), 4 (9.8)

^1^1,185 = invalid or missing; ^2^percentage of total number of hospital stays; ^3^percentage of the category

### Impact of the initial treatment strategy on health care use

Compared with patients who received curative treatment, patients receiving palliative treatment and patients who were given no tumour-directed treatment had significantly higher adjusted IRRs for unplanned hospital stays (IRR 1.31; 95% CI 1.22–1.41, and IRR 1.44; 95% CI 1.30–1.59, respectively) and unplanned specialist outpatient care visits (IRR 1.54; 95% CI 1.42–1.68, and IRR 1.24; 95% CI 1.08–1.42, respectively) (Table 4). Patients with no tumour-directed treatment also had a significantly lower adjusted IRR of planned hospital stays (IRR 0.69; 95% CI 0.58–0.82) and planned specialist outpatient care visits (IRR 0.55; 95% CI 0.51–0.59) compared with patients receiving curative treatment. Compared with patients given curative treatment, patients with palliative treatment had a significantly higher adjusted IRR (1.13; 95% CI 1.09–1.18) for planned specialist outpatient care visits (Table 4).

**Table 4:** The impact of initial treatment strategy on health care use

	Hospital stays, unplanned			
	Unadjusted		Adjusted*	
**Initial treatment**	IRR (95% CI)	p-value	IRR (95% CI)	p-value
No tumour-directed	**1.70 (1.57–1.85)**	**< 0.001**	**1.44 (1.30–1.59)**	**< 0.001**
Palliative	**1.67 (1.59–1.75)**	**< 0.001**	**1.31 (1.22–1.41)**	**< 0.001**
Curative	1.00 (reference)		1.00 (reference)	
	**Hospital stays, planned**			
	Unadjusted		Adjusted*	
**Initial treatment**	IRR (95% CI)	p-value	IRR (95% CI)	p-value
No tumour-directed	**0.67 (0.58–0.78)**	**< 0.001**	**0.69 (0.58–0.82)**	**< 0.001**
Palliative	1.05 (0.98–1.12)	0.179	0.93 (0.84–1.02)	0.133
Curative	1.00 (reference)		1.00 (reference)	
	**Visits, specialist outpatient care, unplanned**			
	Unadjusted		Adjusted*	
**Initial treatment**	IRR (95% CI)	p-value	IRR (95% CI)	p-value
No tumour-directed	1.11 (0.99–1.25)	0.067	**1.24 (1.08–1.42)**	**0.002**
Palliative	**1.53 (1.44–1.63)**	**< 0.001**	**1.54 (1.42–1.68)**	**< 0.001**
Curative	1.00 (reference)		1.00 (reference)	
	**Visits, specialist outpatient care, planned**			
	Unadjusted		Adjusted*	
**Initial treatment**	IRR (95% CI)	p-value	IRR (95% CI)	p-value
No tumour-directed	**0.47 (0.44–0.50)**	**< 0.001**	**0.55 (0.51–0.59)**	**< 0.001**
Palliative	**1.28 (1.25–1.31)**	**< 0.001**	**1.13 (1.09–1.18)**	**< 0.001**
Curative	1.00 (reference)		1.00 (reference)	

*Adjusted for age, sex, M stage and performance status. CI = confidence interval; IRR = incidence rate ratio

### Impact of assignment of a contact nurse on health care use

Patients assigned a CN had a significantly higher adjusted IRR of unplanned hospital stays (IRR 1.29; 95% CI 1.17–1.42), unplanned specialist outpatient care visits (IRR 1.46; 95% CI 1.30–1.64) and planned specialist outpatient care visits (IRR 1.13; 95% CI 1.08–1.19) compared with patients not assigned a CN (Table 5).

**Table 5:** The impact of assignment of a contact nurse (CN) on health care use

	Hospital stays, unplanned			
	Unadjusted		Adjusted*	
**CN assignment**	IRR (95% CI)	p-value	IRR (95% CI)	p-value
Yes	**1.28 (1.16–1.40)**	**< 0.001**	**1.29 (1.17–1.42)**	**< 0.001**
No	1.00 (reference)		1.00 (reference)	
	**Hospital stays, planned**			
	Unadjusted		Adjusted*	
**CN assignment**	IRR (95% CI)	p-value	IRR (95% CI)	p-value
Yes	1.13 (0.99–1.28)	0.076	1.08 (0.94–1.23)	0.288
No	1.00 (reference)		1.00 (reference)	
	**Visits, specialist outpatient care, unplanned**			
	Unadjusted		Adjusted*	
**CN assignment**	IRR (95% CI)	p-value	IRR (95% CI)	p-value
Yes	**1.42 (1.27–1.59)**	**< 0.001**	**1.46 (1.30–1.64)**	**< 0.001**
No	1.00 (reference)		1.00 (reference)	
	**Visits, specialist outpatient care, planned**			
	Unadjusted		Adjusted*	
**CN assignment**	IRR (95% CI)	p-value	IRR (95% CI)	*p*-value
Yes	**1.18 (1.13–1.24)**	**< 0.001**	**1.13 (1.08–1.19)**	**< 0.001**
No	1.00 (reference)		1.00 (reference)	

*Adjusted for age, sex, M stage, performance status and treatment strategy. CI = confidence interval; IRR = incidence rate ratio

## Discussion

To the best of our knowledge, this is the first population-based cohort study that has described the impact of initial treatment strategy on health care use among patients with gastric and oesophageal cancer from the time of treatment decision until death. While previous studies have shown that patients receiving treatment with a curative intent have higher odds of hospitalization in the final months of life compared with patients with a palliative treatment goal [23, 24], this study provides novel insight into the impact of treatment strategy on both planned and unplanned health care use.

The results showed that patients who received tumour-directed treatment had higher rates of planned health care than did patients with no tumour-directed treatment (Table 4). This is in line with previous research that has shown that outpatient care and hospitalizations are particularly common among patients who undergo oncology treatment [30]. One explanation may be that those who undergo tumour-directed treatment are offered planned appointments for the actual treatment but also for disease monitoring and health support to a larger extent compared with those not receiving such treatment. Regular planned health care is likely to facilitate proactivity and care continuity – which are factors that are known to lower the risk for acute admissions to hospital [31–33]. The higher rate of planned care among those with tumour-directed treatment could also be a reflection of the fact that their care is driven by medical treatment of disease and interventions, meaning that the specialized part of the health system is designed to diagnose and treat, rather than prevent, illness or health problems.

One notable aspect of the results is that those with a palliative treatment strategy and those with no tumour-directed treatment used more acute health care than did patients with a curative treatment strategy (Table 4). This is in line with results from previous studies that identified high use of unplanned health care among patients with incurable cancer [17, 18, 34]. Studies by Craigs et al. (2018) and Grande et al. (2002) showed that regular contact with an oncology specialist was associated with increased access to both community and hospital-based palliative care and referral to palliative home care [35, 36]. Palliative care is perhaps especially important among patients with incurable oesophageal and gastric cancer, with regard to their poor prognosis and rapidly deteriorating health condition, and barriers to access services are associated with increased use of unplanned health care [37]. The results suggest that patients with incurable gastric and oesophageal cancer may need closer monitoring and follow-up. However, persons with a palliative treatment strategy had a higher rate of unplanned health care compared with patients with a curative treatment strategy, although the two groups had a similar rate of planned hospital care (Table 4). Previous research has found that acute hospitalizations and frequent ED use are particularly common among patients treated with palliative chemotherapy [34] as a result of both chemotherapy-related side effects and problems associated with progressive disease [38]. In this present study, no data on primary care utilization were available, but research has demonstrated that patients with high utilization of one care provider also have high utilization of other health services [39]. However, if the provided health care service had been optimally tailored and coordinated this would reasonably have led to a lower rate of unplanned health care.

With regard to the rapid disease progression and the fast onset of symptoms, unplanned health care may, on the other hand, be inevitable in a palliative stage of oesophageal and gastric cancer. However, the patients’ reliance on acute hospital care may indicate a lack of alternatives. It is known that a lack of timely access to primary care is associated with ED use [40, 41]. A study by Delgado-Duay et al. (2015) of a population of advanced-stage cancer patients reports that over one-fourth of ED visits could have been avoided by proactive support [42]. In this present study no information was available to determine whether the unplanned health care could have been avoided by proactive efforts addressed in a planned manner elsewhere.

A proactive approach requires coordination and communication between caregivers. It could be argued that preventive interventions should be facilitated in primary care and that fundamental palliative care should be provided by the primary care provider – this may, however, be challenging if the patient has several complex needs in the early stage of disease. A study by Beernaert et al. (2014) indicates that family physicians do not systematically assess non-acute care needs in patients with advanced-stage disease and more often pay attention to palliative care needs in the terminal phase [43]. Specialized health care services could provide an opportunity to proactively identify health care needs and coordinate interventions. A study by Snyder et al. (2008) showed that patients who receive care, both from oncology specialists and from their primary caregiver, received more preventive interventions, compared with patients receiving regular care from their primary care provider alone [44]. Proactive symptom management and instant access to specialized health care service may be especially important – and inevitable – in the care of patients with oesophageal and gastric cancer. The disease causes severe and often acute problems with dysphagia, bleeding and obstruction, which requires acute surgical interventions to alleviate cancer-related symptoms [45]. Regular follow-up care and easily accessible specialized health care may be essential if health care providers are to have a chance to proactively identify and address care needs.

The ASCO (2017) recommends integration of a palliative care approach shortly after the advanced cancer diagnosis to ensure proactive and coherent health care [12]. They state that acute hospitalizations and repeated ED visits are not consistent with high-quality palliative care and should, as far as possible, be avoided by means of anticipatory symptom management and advanced care planning [16]. This is also in line with patient preferences as the majority of patients with advanced cancer prefer to be cared for and die at home, rather than in a hospital care setting [46]. Frequent hospitalization can cause great distress to patients and their families [47, 48], and the acute hospital setting is considered suboptimal with regard to palliative care, in terms of insufficient symptom management, family support and end-of-life communication [49]. Palliative care, integrated early in the disease trajectory, has been related to reduced risk for unplanned hospital care [50–52]. The higher rates of unplanned health care among patients with a palliative treatment strategy and no tumour-directed treatment in our study suggest that a more proactive approach and enhanced care planning may be needed. It is also important that those who are not considered curative receive planned, monitored care and preventive interventions.

According to a statement in Sweden’s national cancer strategy, people with a cancer diagnosis should be offered a permanent care contact in order to obtain more coherent and effective care [15]. The CN in Swedish cancer care has a similar role as a nurse navigator, providing advice, coordinating care and mediating contact with other health care professionals, although there can be a wide variation in these nurses’ scope of practice [53]. Our findings showed that patients assigned a CN had higher rates of unplanned care and planned outpatient care visits, compared with patients not assigned a CN (Table 5). A study conducted by Gordon et al. (2019) reports that patients assigned a nurse navigator had more hospital admissions and ED visits, than did patients without a nurse navigator, while another study has shown inconsistent findings on the relationship between CNs and health care use [54, 55]. It is possible that CNs enhance access to health care and this could be because of CNs’ mandate to facilitate health care access within their own organization. Another possible explanation could be that patients who lack a CN are more likely turn to their primary care provider. However, this needs to be further investigated, since this study did not include data on primary care use.

A major strength of this study is the large sample, comprising patients from different geographical areas representative of Sweden’s population. The results were based on data from the NREV, which has an accuracy of 91.1% and a completeness rate of 95.5% among diagnosed patients [25], and the NPR, which has a completeness rate of 99 and 87% for inpatient and outpatient care, respectively [29].

Despite this, some limitations need to be considered. In total, information on CN assignment from the NREV was missing for 1380 patients. Missing data on this variable are related to the fact that a CN assignment did not fully become standard practice until the introduction of the standardized care pathway in 2015. This was also confirmed by analysis showing a higher rate of missing data on CN assignment before 2015 compared with after (5% v 83%). Dropout analysis showed that patients who lacked data on CN assignment were significantly younger (mean age 70.32 v 72.63; *p* **<** 0.001), had a longer survival time (median 12 months v 4 months; p **<** 0.001) and had a lower proportion of distant metastases (24.3% v 50.8%). These differences can be related to the fact that most patients for whom data on CN assignment were available were diagnosed in 2015 and, since we included patients who died between 2014 and 2016, they were likely to have a short survival time. Because of these differences, the findings cannot be generalized to the entire cohort. Another limitation is that we do not have information on the exposure-dose i.e. the frequency of contacts and exact time period the patients were exposed to a contact nurse. The result has to be interpreted with this in mind. Further studies that focus on the extent and content of CN interaction would therefore be interesting.

Patients were categorized based on *planned* treatment, and some patients may not have been treated accordingly. In particular, this concerns patients treated with curative intent who progressed to advanced disease after the initial treatment decision had been made. However, subsequent decisions to resume the intended curative treatment would minimize the differences between the three groups and consequently make detection of the observed effect less likely. The analyses were adjusted for age, sex, M stage and performance status, but because of the observational design, unmeasured confounding cannot be completely excluded.

Another limitation is the lack of information on primary health care, which was not available from the NPR. It is possible that patients with no tumour-directed therapy received planned care from their primary care provider to a higher extent, compared with patients treated with curative intent. Future studies should investigate health care utilization in primary care.

In conclusion, the results of the current study show that a palliative treatment strategy and no tumour-directed treatment were associated with higher rates of unplanned health care compared with a curative treatment strategy. This finding may be a sign of insufficient support for a vulnerable group of patients and may highlight the need for early and proactive palliative care.

## Data Availability

Some restrictions will apply. Data cannot be shared publicly because of regulations in the Swedish Data Protection Act (2018:218; 2019; 219) and Ethical Review Act (2003:460), data are available from the holders of the registers; NREV (Jan Johansson, Jan.johansson@med.lu.se) for researcher who meet the criteria for access to confidential data.
